# Precision renal medicine: a roadmap towards targeted kidney fibrosis therapies

**DOI:** 10.1186/s13069-015-0033-x

**Published:** 2015-09-01

**Authors:** Michael Zeisberg, Elisabeth M. Zeisberg

**Affiliations:** Department of Nephrology and Rheumatology, University Medical Center Göttingen, Georg August University, Robert Koch Str. 40, 37075 Göttingen, Germany; Department of Cardiology and Pneumology, University Medical Center Göttingen, Georg-August University, Göttingen, Germany; German Center for Cardiovascular Research (DZHK), Robert Koch Street 40, Göttingen, Germany

**Keywords:** Fibrosis, Precision medicine, Epigenetics, Liquid biopsy, Clinical trial

## Abstract

Based on extensive pre-clinical achievements over the past decades, it appears to be due time for a successful clinical translation in the renal fibrosis field—but what is the quickest road to get there? In light of the recent launch of the Precision Medicine Initiative and success of molecularly informed drugs in oncology, we here discuss what it may take to bring molecularly targeted anti-fibrotic to clinical use in chronic progressive kidney disease.

## Review

### Introduction

As the era of precision medicine begins to become reality in oncology, “aiming to classify individuals into subpopulations that differ in their susceptibility to a particular disease, in the biology and/or prognosis of those diseases they may develop, or in their response to a specific treatment”, and to utilize such comprehensive knowledge for molecularly informed therapies, there still is no therapy available to inhibit fibrogenesis that underlies progression of chronic kidney disease as yet. With regard to the recently proposed Precision Medicine Initiative in the USA with its two main components of “a near-term focus on cancers and a longer-term aim to generate knowledge applicable to the whole range of health and diseases” (including chronic kidney disease and fibrosis), we here review how modern cancer medicine evolved and discuss how such knowledge gained in the cancer field can be utilized to kick-start the era of precision renal medicine and to implement molecularly informed anti-fibrotics into clinical use.

### Evolution from nitrogen mustard chemotherapy to molecular targeted cancer therapy

The evolution of cancer therapies was kick-started by initial studies by Gilman and Goodman linking nitrogen mustard to regression of lymphoid tumors and clinical studies by Farber documenting remission of acute lymphoid leukemia upon administration of the folate antagonist aminopterin [1–3]. While these initial chemotherapies were considerably toxic, they served as proof-in-principle for effective cancer therapies and eventually led to the concept of potential cure of cancer [4]. For 50 years, relatively unspecific and toxic substances, which primarily aimed to kill actively dividing cells, remained the mainstay of cancer therapies [5]. The new era of molecular targeted therapies (use of drugs or other substances that block the growth or spread of cancer by interfering with specific molecules) started with approval of imatinib (Gleevec) for clinical use in Philadelphia chromosome-carrier CML patients [6]. The Philadelphia chromosome refers to a reciprocal transclocation in which a region of chromosome 9 encoding for the Abl tyrosine kinase is fused to the BCR (breakpoint-cluster-locus), resulting in a constitutively active BCR-Abl tyrosine kinase fusion protein which ultimately provides a constant proliferative signal in cancer cells [6]. Imatinib is a tyrosine kinase specific inhibitor specifically designed to fit the BCR-Abl ATP-binding site of the protein created by the BCR-Abl translocation enabling for the first time specific cancer therapy by targeting a causal molecular target [7]. Today, there are already more than 75 FDA approved targeted cancer therapies, which are being used based on presence of respective targets within malignant cells (http://www.cancer.gov). With still evolving molecular analytical tools and rapidly increasing numbers of target-specific drugs, the cancer field is currently in flux: It is becoming increasingly obvious that cancers, which were previously classified as single entity based on their tissue of origin and localization, can be grouped into distinct subtypes due to their molecular signatures [8–10]. Such molecular signatures can serve as basis for individually stratified molecularly targeted therapies, whereas traditional classifications were used to stratify rather unspecific chemotherapies [8, 11]. Furthermore, it is becoming evident that based on their molecular signatures and disease drivers, tumor subtypes of one organ origin differ substantially from another but can be almost identical to tumor subtypes from distant organs [12]. For example, bladder cancers could be classified into subgroups, which remarkably were almost indistinguishable from either lung adenocarcinomas or squamous cell cancers of the head and neck [12]. These newly found molecular characteristics have important implications for cancer therapies: For example, in 60 % of melanoma patients, the B-Raf proto-oncogene, serine/threonine kinase (BRAF) is mutated [13]. Those patients respond to the BRAF inhibitor vemurafenib; BRAF-negative patients do not [13]. However, patients with hairy cell leukemia with BRAF mutations often also respond to vemurafenib therapy, exemplifying applications of molecular diagnostics and targeted therapies [14]. Overall, such “genomically informed cancer therapies” are proving far superior over non-targeted therapies and have prompted calls for reclassification of cancers based on molecular profiles and targets instead of being based on traditional histopathology and origin—and opened the door for the Precision Medicine Initiative [15].

As the cancer field is moving from histopathological analysis to molecular profiling of cancer, not only traditional histopathology-based tumor classifications are being challenged; the benefits of tumor biopsies per se is also under scrutiny and analysis of cell-free DNA (cfDNA) as “liquid biopsy” is increasingly being recognized as complementation or even alternative to standard tumor biopsies [16]. Presence of fragmented cell-free DNA in the blood long has been known [17]. Clinical utilization of cfDNA was first adapted in pre-natal diagnostics, where fetus-derived cfDNA fragments within maternal blood are used to determine gender and developmental disorders such as Down’s syndrome of the fetus [18]. Studies from the cancer field established that cfDNA obtained from blood represented all DNA modifications present within primary tumors and metastases: point mutations, rearrangements, amplifications, aneuploidy, and DNA methylation [19]. With current technologies, sensitivity of cfDNA analysis is reaching almost 100 % and, due to accounting for tumor heterogeneity, outperforms tumor biopsies with regard to molecular profiling. Because blood sampling is minimally invasive, analysis of cfDNA appears particularly primed for longitudinal analysis of tumor burden and monitoring of treatment response. In this regard, analysis of cfDNA clearly outperformed protein biomarkers and imaging techniques [19, 20]. Taken together, the cancer field has evolved dramatically over the past two decades, opening the door for precision medicine.

### Obstacles and possible solutions for renal fibrosis

As compared to the cancer field, the one initiating clinical study providing proof-in-principle that fibrosis can be reversed in patients is still lacking. However, founded on strong evidence from murine studies, the concept of irreversibility of chronic kidney disease has given way to increasing confidence that renal fibrosis is a treatable target in principle and that possible regression of fibrosis would translate into preservation of kidney function. The new era of anti-fibrotics in the kidney was initiated by the report of reversal of experimental chronic kidney disease in 2003 [21] . While several clinical trials to test efficacy of anti-fibrotics in chronic kidney disease are currently underway [22], several obstacles need to be overcome to enable a precision anti-fibrotic renal medicine, possibly by benefitting from the recent advances made in the cancer field:

### Possibilities for molecularly informed anti-fibrotic therapies

Majority of current anti-fibrotic trials in chronic kidney disease are not only done without molecular profiling of enrolled patients; they are also done without kidney biopsy (www.ClinicalTrials.gov). This can be mostly attributed to the fact that in patients with presumed diabetic nephropathy and hypertensive nephrosclerosis, kidney biopsy is not done for diagnostic purposes as diagnostic insights do not outweigh procedural risks. In analogy to the cancer field, clinical trials conceptually follow chemotherapy designs in the cancer field during the pre-precision medicine area under the assumption that one anti-fibrotic drug could be beneficial to all patients with chronic kidney disease (the concept of “one-size-fits-all” therapies). Notably, this is done with candidate compounds which specifically target select molecular mechanisms such as TGF-beta signaling, CTGF, or the chemokine receptor 2, which were derived through molecular target identification and which fulfill requirements for “molecularly informed targeted therapies”—only that diagnostic molecular information of candidate patients are not being assessed [22].

One reason for such strategy is likely the existing concept of common kidney fibrosis pathways [23, 24]. Arguments for such concept are obvious: Histopathological studies revealed that the extent of tubulointerstitial fibrosis is the single best predictor of kidney function, even in primary glomerular diseases [24, 25]. Because of its uniform appearance, the obvious assumption of a uniform kidney fibrosis pathway underlying all chronic progressive nephropathies evolved [24, 26–34]. The concept of a common fibrosis mechanism was further corroborated by overwhelming experimental evidence for a role of transforming growth factor beta1 in renal fibrogenesis: Inhibition of TGFβ-inhibited fibrosis in murine models commonly used in kidney fibrosis research [35–38], TGFβ was found to induce cellular events typically associated with fibrosis in commonly used cell lines [39, 40], and TGFβ was also implied as principal pro-fibrotic mediator in other organs such as the skin, liver, and lung [41–43]. Based on this overwhelming body of evidence, TGFβ inhibition was identified as lead anti-fibrotic target and three independent clinical trials with TGFβ inhibitors have been initiated in the kidney (for review see). However, proof of envisioned efficacy is still lacking (Dr. James Voelker of Eli Lilly and Co. reported at the ASN Kidney Week 2014 that anti-TGFβ1 therapy in patients with advanced diabetic nephropathy with a humanized anti-TGFβ1 antibody LY2382770 in a phase II dose-ranging study was terminated early due to efficacy futility [22]).

In hindsight, this may not be surprising. As TGFβ was identified as a major mediator of common kidney fibrosis pathways, controlled clinical studies which analyzed utility of circulating or urinary TGFβ as biomarker for chronic progressive kidney disease did not reveal a prognostic value [44, 45]. Retrospectively, this may have been due to the fact that there are patients in which TGFβ is not involved—simply put that kidney fibrosis pathways are less common than previously thought. In this regard, a previous study reported that experimental renal fibrosis also occurs when TGFβ signaling is inactive (due to lack of thrombospondin)—it just takes longer to evolve [46]. Importantly, such TGFβ-independent tubulointerstitial fibrosis differed in cellular and extracellular composition—even though such differences were not detected by standard histopathological analysis of the relative interstitial volume [46].

Cancer biology taught three principal aspects: That among cancers which are being grouped as one entity according to established classifications, subgroups exist which can be classified according to their underlying oncogenic pathways, that these oncogenic pathways are disease drivers within cancers of different entities at different organs, and that with regard to molecularly informed targeted therapies the molecular signatures are more relevant than traditional classifications. In analogy, recent evidence regarding renal fibrogenesis is suggesting that there are distinct fibrotic pathways which underlie progression of chronic kidney disease (and that these pathways could serve as basis for classification of chronic kidney disease for molecularly informed therapies). Furthermore, evidence is also suggesting that these pathways (i.e., TGFβ signaling) are disease drivers in subtypes of fibrosis across all organs. One should not take the second step before the first step: As molecularly targeted anti-fibrotics are available, it appears reasonable to use them in a molecularly informed fashion and to implement molecular profiling into clinical diagnostics and clinical-trial designs.

### Attraction of a liquid renal biopsy

While kidney tissue is the gold standard for clinical diagnosis and investigational sequencing, kidney biopsies are associated with several limitations per se*.* Percutaneous kidney biopsies are not only an inconvenient, cost-intensive procedure; they often are without impact on clinical outcome (i.e., in context of diabetic nephropathy, hypertensive nephrosclerosis, or acute kidney injury), they are also not without clinical complications (main complications are gross hematuria, transfusion-requiring bleeding, abcesses, and urosepsis and occur at rates between 2 and 4 % in the literature). Furthermore, standard techniques (formalin-fixation and paraffin-embedding) used for histopathological analysis impair current sequencing technologies through DNA crosslinking. Also, tissue heterogeneity is a major limitation of kidney biopsies, as tubulointerstitial fibrosis (and subsequently DNA-, RNA-, and protein-modifications) is not evenly distributed within the diseased kidney and common biopsy-cores are often not representative. To overcome these limitations, less invasive diagnostic tests which most importantly reflect tissue heterogeneity would be highly desirable.

Despite convincing utility of cfDNA analysis for cancer diagnostics, there are obvious limitations to translate such technology to renal medicine: Based on current knowledge, there are no obvious point mutations, rearrangements, amplifications, or aneuploidy involved in progression of renal fibrogenesis. As large cohorts studies to identify genetic polymorphisms which are directly linked to susceptibility for fibrosis progression are underway [47–49], it is clear that even if such traits will be identified, they will not lend themselves for monitoring of disease progression or treatment responses (based on current knowledge, fibrogenesis is not associated with acquisition of de novo somatic mutations or polymorphisms and hence do not lend themselves for longitudinal analysis—unlike in cancer where genetic instability and de novo mutations are a hallmark of cancer progression).

Recent studies suggest that these limitations can be overcome by analysis of epigenetic marks, because epigenetics in general offer the advantage of greater stability over mRNA or protein-based biomarkers (which are more dynamic and subject to greater fluctuation, often providing a snap-shop at time of analysis over a long-term picture which underlies individual progression of kidney fibrosis) but do underlie modifications during disease progression or treatment responses as opposed to genetics [50, 51]. While major epigenetic mechanisms—CpG promoter methylation, histone modifications, and micro RNAs—interact and impact each other, analysis of DNA methylation offers the advantage of being gene target specific by definition, whereas histone modifications and micro RNAs target numerous genes, requiring further complex analyses (if a specific gene promoter is methylated, as possibly detected by methylation-specific PCR, this means that transcription of this specific genes is silenced) [50, 52]. Altered presence of single microRNA causes altered expression of numerous genes, typically requiring additional transcriptional profiling to assess context dependent relevance [53]. Histone modifications are most complex, as those occur at multiple sites within multiple genes, blurring assessment of biological impact of identified modification [54]. In this regard, in context of kidney fibrosis several methylation marks with direct causal consequence to renal fibrogenesis have been identified, similar to what has been revealed by analysis of monozygous twins in the cancer field [50, 55–57]. What is even more appealing is that levels of circulating methylated DNA promoter fragments reflect intrarenal levels of methylated promoter CpG islands of respective genes, providing a cue for a liquid renal biopsy in the future [50]. For example, our group identified that CpG promoter methylation of *RASAL1* (which encodes for an endogenous Ras-GTP inhibitor) causes transcriptional silencing of this gene and contributes causally to progression of renal fibrogenesis [58]. We also identified that levels of *RASAL1* CpG island promoter methylation correlates with extent of tubulointerstitial fibrosis in renal biopsies and with GFR decline [40]. Finally, we reported that levels of circulating methylated DNA promoter fragments reflect intrarenal levels of methylated promoter CpG islands of RASAL1 and also correlated with successful de-methylating therapies in mice [50]. While more work needs to be done to identify disease relevant threshholds of RASAL1 methylation, the use RASAL1-methylation stratified use of Ras-GTP inhibitors could serve as one example of how a n epigenetic biomarker-informed molecular therapy could be envisioned (just like methylated fragments of TGF-β and its receptors could be taken as strong indication not to use TGF-β inhibitors).

## Conclusions

Based on extensive pre-clinical achievements over past decades it appears to be due time for successful clinical translation in the renal fibrosis field—but what is the quickest road to get there? With the bar being set high by the US Food and Drug Administration (the FDA currently accepts halving of glomerular filtration rate (GFR)—and in certain circumstances 40 % over 2–3 years, as a surrogate end point clinical trials of CKD progression [59]), it may be time well-spent to re-think current clinical trial strategies to increase likelihood of success (and to avoid costly avoidable failures). Based on the substantial advances made in the cancer field, which culminated in molecularly informed therapeutics and launch of the precision medicine area, we believe that it may be wise to learn from the growing pains of oncology and utilize such experience for renal fibrosis trial design. In this regard, there seems to be a mismatch between the specific molecularly targeted anti-fibrotics which are being tested and the relatively uninformed trial designs which are being used.

Per current thinking, patients diagnosed with diabetic nephropathy and or hypertensive nephrosclerosis or FSGS at CKD stages 2–3 are regarded as attractive study population, because such patients promise rapid-enough disease progression to obtain results with relatively short observation time (2–3 years), and because they are common enough to recruit sufficient patient numbers for adequately powered trials (based on standard metrics). Obvious drawback of such design is that patients mostly do not undergo kidney biopsy at study entry (especially in case of diabetic nephropathy and nephrosclerosis) and that there is no knowledge if the targeted disease mechanism is even active. Bluntly putwith the example of TGF-β inhibition: when TGF-β signaling is not active, TGF-β inhibition cannot work. In other words in analogy to the cancer field—current renal fibrosis trials follow the trial design of when nitrogen mustard was used, neglecting possibilities of molecular stratification. It just seems plausible that the likelihood of therapeutic success is higher when the molecular target of a moleculalry targeted therapy is active in a respective patient and that it would be of benefit to enrich such patients in a study cohort by analyzing such targeted pathway at the time of patient recruitment.

One reason for such neglect might be the concept of a uniform fibrosis pathway, which conceptually justifies aforementioned “one-size-fits-all” therapeutic strategies. The promise of a common pathway which drives fibrosis in all organs (and thus causally contributes to 30 % of mortality world-wide) has obvious charms; but evidence is increasing that this may have been an over-simplified view. While such concept enhanced interaction of fibrosis research across all disciplines, these interactions revealed that fibrosis across organs is similar, albeit with different flavors and nuances [60]. And similarly, it appears plausible that—while mechanisms of fibrosis are similar in individual patients, there may be differences which pathway is the causal driver in such patients. Difficulties to establish known pro-fibrotic mediators (such as TGF-β) reliable biomarkers of CKD, point towards such thinking. Again in analogy to advances in the cancer field disease, progression in a patient with diabetic nephropathy and high expression of TGF-β may be more similar to a patient with interstitial nephrits and high TGF-β levels as compared to a patient with diabetic nephropathy and low TGF-β expression. And in further analogy, it appears plausible that the fibrosis in patients with interstitial nephritis and high eosionophiluria is more similar to the fibrosis in patients with idiopathic pulmonary fibrosis and high eosinophil count within bronchial fluid than to the fibrotic process in patients with hypertensive nephrosclerosis, particularly from a therapeutic point of view. Introduction of CKD grading and staging criteria may have been a first step towards a molecular pathway-based classification, and in this regard emerging big data should be utilized to identify specific subsets of patients (as opposed to solely focusing on common denominators) and we believe that analysis of epigenetic modifications in fluids such as blood and urine may have the highest utility to identify such patient subsets. While the concept of a personalized medicine has been known for a while, launch of the Precision Medicine Initiative and obvious difficulties to translate pre-clinical advances to clinical practice in nephrology should serve as exclamation marks to nudge the renal fibrosis field to finally adopt such thinking.
